# Dual Role of Surface Hydroxyl Groups in the Photodynamics and Performance of NiO-Based Photocathodes

**DOI:** 10.1021/jacs.2c04301

**Published:** 2022-06-08

**Authors:** Kaijian Zhu, Sean Kotaro Frehan, Guido Mul, Annemarie Huijser

**Affiliations:** PhotoCatalytic Synthesis Group, MESA+ Institute for Nanotechnology, University of Twente, P.O. Box 217, AE Enschede 7500, the Netherlands

## Abstract

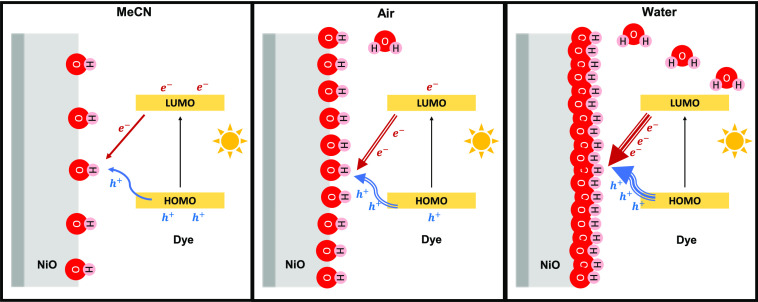

Photoelectrochemical (PEC) cells containing photocathodes based on functionalized NiO show a promising solar-to-hydrogen conversion efficiency. Here, we present mechanistic understanding of the photoinduced charge transfer processes occurring at the photocathode/electrolyte interface. We demonstrate via advanced photophysical characterization that surface hydroxyl groups formed at the NiO/water interface not only promote photoinduced hole transfer from the dye into NiO, but also enhance the rate of charge recombination. Both processes are significantly slower when the photocathode is exposed to dry acetonitrile, while in air an intermediate behavior is observed. These data suggest that highly efficient devices can be developed by balancing the quantity of surface hydroxyl groups of NiO, and presumably of other p-type metal oxide semiconductors.

## Introduction

Development of highly efficient solar cells and photoelectrochemical (PEC) cells is essential to facilitate the energy transition and mitigate climate change. Previous research efforts to improve the performance of solar cells or PEC cells include doping of metal oxide semiconductors,^1−3^ evaluation of combinations of light absorbers and catalysts,^4−6^ and the introduction of hole or electron transport layers,^7−9^ or surface passivation layers.^10,11^ However, the efficiency of PEC cells, in particular, is still unsatisfactory; especially, the realization of effective dye-sensitized photocathodes remains a challenge.^12−15^ Dye-sensitized photocathodes consist of dye molecules and catalysts adsorbed on a p-type metal oxide semiconductor. The dye absorbs the light and generates the electrons and holes. Different from electrodes based on a metal oxide semiconductor only, in which light absorption and charge separation occur in the same material, the light-induced charge separation in a dye-sensitized photocathode occurs via hole injection from the dye into the semiconductor, followed by electron transfer to the catalyst.

A major challenge in both types of photoelectrodes is the prevention of fast charge recombination processes leading to major efficiency losses.^16−18^ While the surface structure of the metal oxide is thought to play an important role in the determination of the charge separation efficiency, the mechanistic understanding is limited, and in particular, the role of (surface adsorbed) water and hydroxyl groups is unclear. Li et al. and Luo et al. reported that surface-bound OH species on hematite (Fe_2_O_3_) used in PEC cells serve as a hole collection and transfer mediator, accelerating charge collection and enhancing the photocurrent.^19,20^ In contrast, Iandolo et al.^21^ concluded that an OH-terminated hematite surface acts as a hole recombination center, and Jiang et al. and Li et al. claimed that the surface hydroxyl groups on TiO_2_ function as charge recombination centers.^22,23^

Inconsistent results on the role of hydrated semiconductor surfaces are observed not only in solar water splitting but also in the research on dye-sensitized solar cells (DSSCs). The performance of the most popular p-type semiconductor, NiO, is known to be highly sensitive to sometimes only minor differences in the preparation method.^24^ Surface states associated with hydroxyl groups and oxygen sites of the NiO may introduce intra-band-gap states and cause charge recombination.^25^ Therefore, passivation of surface states seems to be a promising method to reduce charge recombination. Cahoon et al. applied target atomic deposition of Al on NiO and observed an improvement in the solar cell efficiency in an aqueous electrolyte.^26^ However, when investigating the performance in dry acetonitrile for comparison, the passivated NiO showed the lowest short-circuit current and efficiency. Tian et al. reported similar results, the solar cell (in an acetonitrile electrolyte) showed a surprisingly low short-circuit current after passivating 72% of the surface states via atomic layer deposition of Al_2_O_3_, which was attributed to inefficient dye regeneration.^27^

The surface of NiO is known to be highly complex.^28−30^ Surface adsorbates such as O_2_ will promote H_2_O adsorption and dissociation on the NiO surface, either at a defect site or at a regular site, depending on the facet.^31−34^ The work function of NiO is known to linearly drop with the coverage of water molecules.^35^ The conductivity of NiO films has recently been reported to be determined by surface states rather than by bulk properties.^27^ Furthermore, the conductivity of NiO films changes dramatically after aging in a natural atmosphere.^36^ A possible reason is a surface change due to different coverages by H_2_O and Ni–OH. In addition, NiO has different surface conditions in aqueous and aprotic electrolytes.^37^

Considering the high surface sensitivity of NiO, the photoinduced interfacial charge carrier dynamics of a NiO-based DSSC, PEC cells or other devices, likely vary in different electrolytes. However, most modern characterization techniques are not in situ, and the characterized surface condition is hence not the same as that in working conditions. In this work, we unravel for the first time the role of surface hydroxyl groups in the interfacial charge dynamics of photosensitized nanoporous NiO using time-resolved photoluminescence (PL) and femtosecond transient absorption (TA) spectroscopy under in situ conditions. The NiO is functionalized with P1 [4-(*bis*-4-(5-(2,2-dicyano-vinyl)-thiophene-2-yl)-phenyl-amino)-benzoic acid] dye molecules, which absorb well below 600 nm. Sun and co-workers designed this dye for the photosensitization of NiO and other p-type semiconductors.^38^ P1 is equipped with a COOH anchoring group and has a delocalized highest occupied molecular orbital (HOMO) and a lowest unoccupied molecular orbital (LUMO) localized far away from the anchoring group, which supports light-induced charge separation. We observe that photoinduced hole injection from the P1 dye into NiO is very slow in dry acetonitrile (the surface containing small amounts of surface H_2_O and Ni–OH) but ultrafast in phosphate buffer solution (PBS, pH 7, i.e., ample-surface H_2_O and Ni–OH). On the contrary, the reduced dye formed via photoinduced hole injection into the NiO decays much slower in dry acetonitrile than in air and especially in PBS due to slower charge recombination. We demonstrate that these effects are due to the two functions of surface hydroxyl groups: increasing the hole injection rate from the dye into NiO and promoting hole accumulation, thereby increasing the charge recombination rate. This work emphasizes the importance of balancing the amount of surface hydroxyl groups of NiO-based photocathodes, which likely explains the contradictory results reported in earlier publications.

## Results and Discussion

### Photoelectrode Characterization

**Figure 1:**
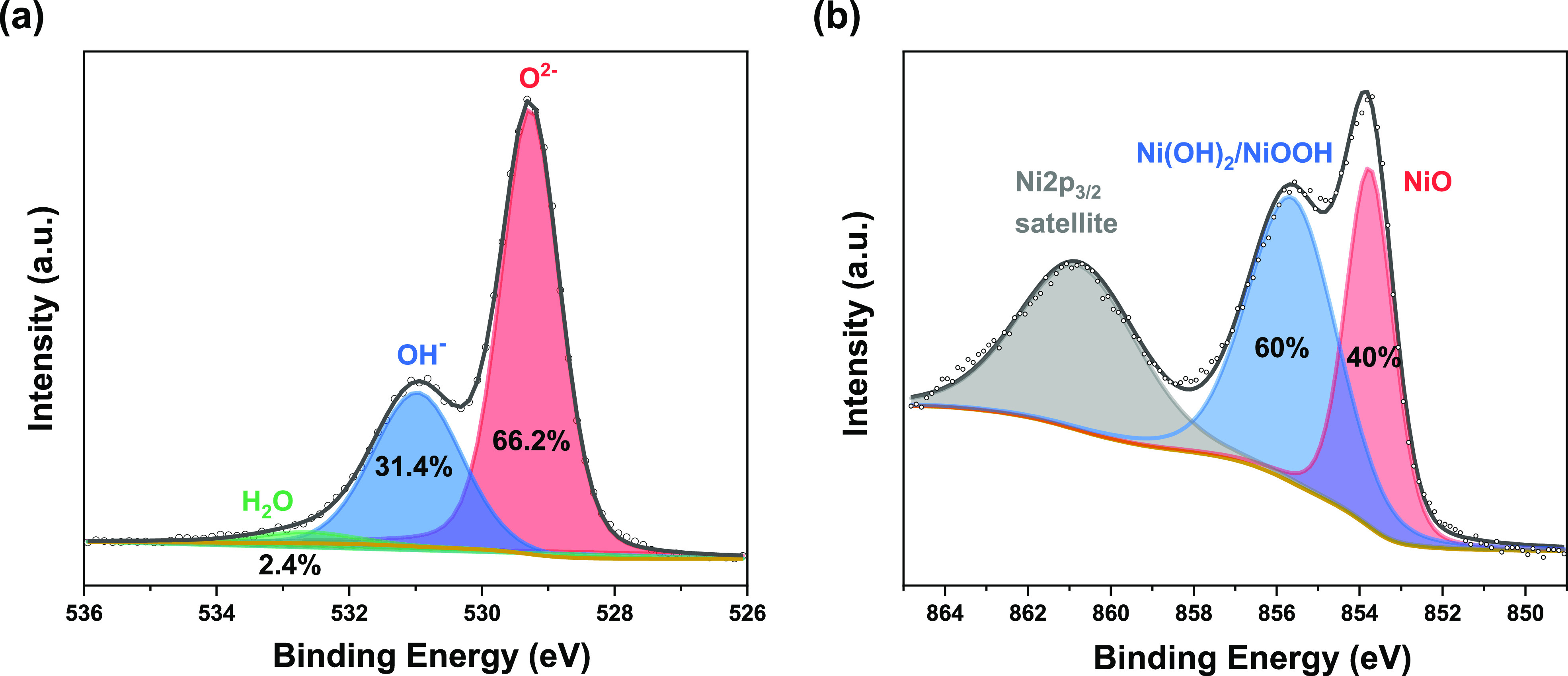
XPS spectra of the NiO film and deconvolution: O 1s (a) and Ni 2p_3/2_ (b) bands.

Nanoporous NiO films with a thickness of ca. 230 nm (see Figure S1 of the Supporting Information for scanning electron micrographs) were prepared as described in the Experimental Section (see Supporting Information). The X-ray photoelectron spectroscopy (XPS) results are shown in Figure 1. X-ray diffraction data of the bare NiO layers and UV–vis absorbance spectra of bare and P1-photosensitized NiO layers are provided in Figure S2 of the Supporting Information. Deconvolution of the XPS O 1s spectrum (Figure 1a) confirms three different O species for the NiO surface. The peaks at around 529.3, 531.0, and 532.9 eV are assigned to O^2–^, Ni–OH, and surface-adsorbed H_2_O, respectively.^39,40^ The ratio of O^2–^ to OH^–^ surface termination is around 2:1. The assignment of the Ni 2p_3/2_ peak (Figure 1b) is nontrivial due to the complexity of the 2p spectra.^41^ Here, we only assigned the peak around 853.8 eV to Ni^2+^ since the peak around 855.7 eV is likely a mixture of Ni^2+^ and Ni^3+^, as in Ni(OH)_2_ and NiOOH, respectively.^39,42,43^ The lack of a peak at a low binding energy of around 851.9 eV indicates the absence of metallic Ni in the film.^42^ Apparently, the surface of as-prepared NiO contains a large amount of surface hydroxyl groups, even in the high vacuum conditions required for XPS analysis.

### Surface Hydroxyl Groups Assist Photoinduced Hole Injection

**Figure 2:**
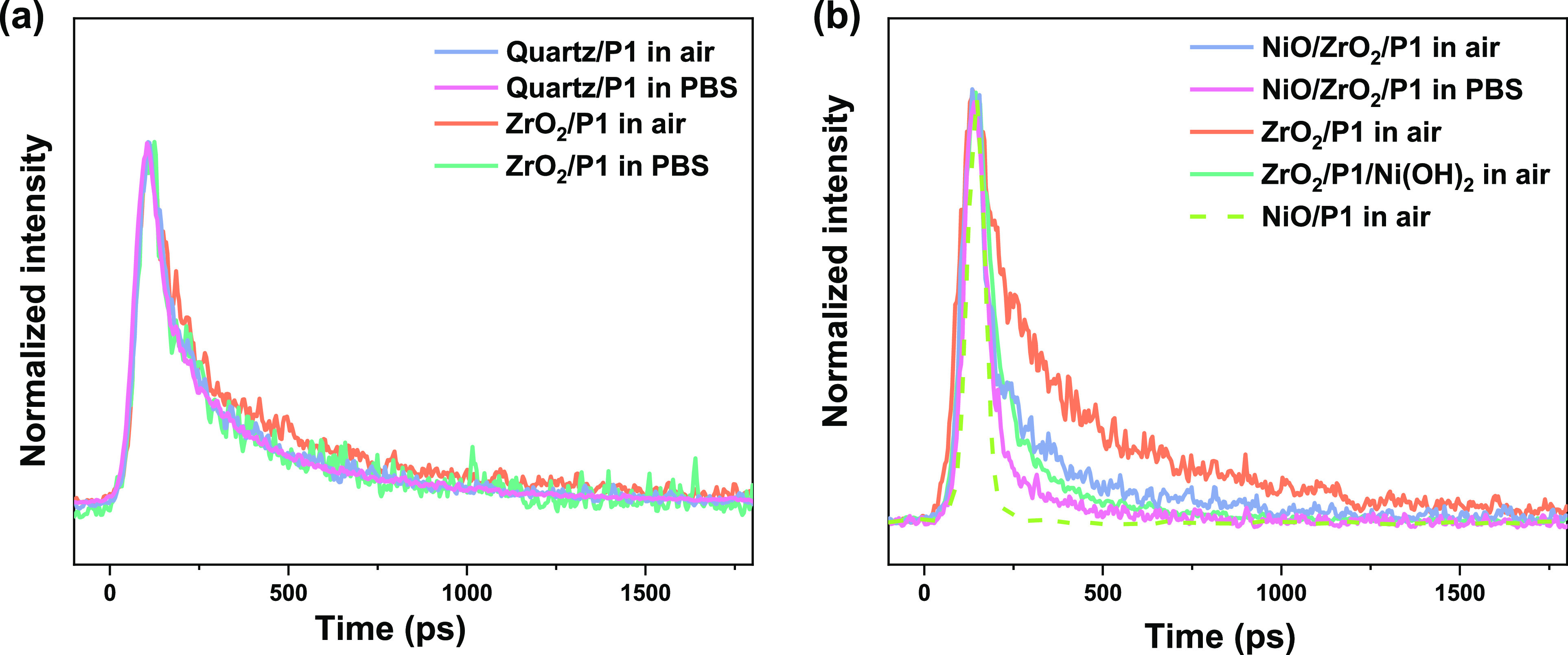
Normalized PL decay at 670 nm of the P1-sensitized layers recorded at 532 nm excitation. (a) Experimental conditions have little effect on the PL decay in the absence of NiO, while in the presence of NiO (b) experimental conditions alter the decay. Note that in one sample, the light absorption layer was terminated by a Ni(OH)_2_ layer to consolidate the effect of surface OH^–^ groups.

Time-resolved PL measurements at 532 nm excitation were carried out to study the excited-state dynamics of the P1 dye used for photosensitization on various substrates in different environments. Figure 2a shows the normalized PL decay at 670 nm of P1 on quartz and on ZrO_2_ in air and in PBS (pH 7, the commonly used electrolyte for PEC H_2_ evolution). All exhibit the same PL decay, showing no additional quenching channel is formed via either photoinduced hole injection into the ZrO_2_ or electron injection into the PBS. Conversely, the PL decay is extremely fast for P1 on NiO (Figure 2b), likely due to the photoinduced hole injection from P1 into NiO, usually occurring in less than 10 ps.^44^ When a ZrO_2_ layer is introduced between the NiO surface and the P1 dye, the PL decay changes. Since ZrO_2_ is an insulator and has a deeper valence band position than NiO and P1,^45,46^ it likely inhibits (part of) the photoinduced hole injection from P1 into NiO, resulting in slower PL quenching than for NiO/P1. Interestingly, when PBS is present, the PL quenching is accelerated. As the PBS has no significant effect on either ZrO_2_ or the P1 dye (Figure 2a), a likely reason is a change in the structure of the NiO surface, affecting the hole injection dynamics. In an aqueous electrolyte, water molecules are known to be easily adsorbed on the NiO surfaces and form Ni(OH)_2_.^38^ Therefore, it is reasonable to assume that the presence of a larger quantity of surface hydroxyl groups is the reason for the decrease in the PL lifetime. An additional thin layer of Ni(OH)_2_ was deliberately deposited on the top of the ZrO_2_/P1 layer following literature procedures to consolidate this hypothesis.^47^ The PL decay of ZrO_2_/P1/Ni(OH)_2_ (Figure 2b, green line) indeed shows a faster PL decay compared to ZrO_2_/P1. This accelerated PL decay is almost similar to NiO/ZrO_2_/P1 in PBS, strongly suggesting that hole injection by the excited P1 dye into Ni(OH)_2_ is responsible for the PL quenching. The different amounts of surface hydroxyl groups might also be the reason for the variety in hole injection rates reported in the literature.^48^

**Figure 3:**
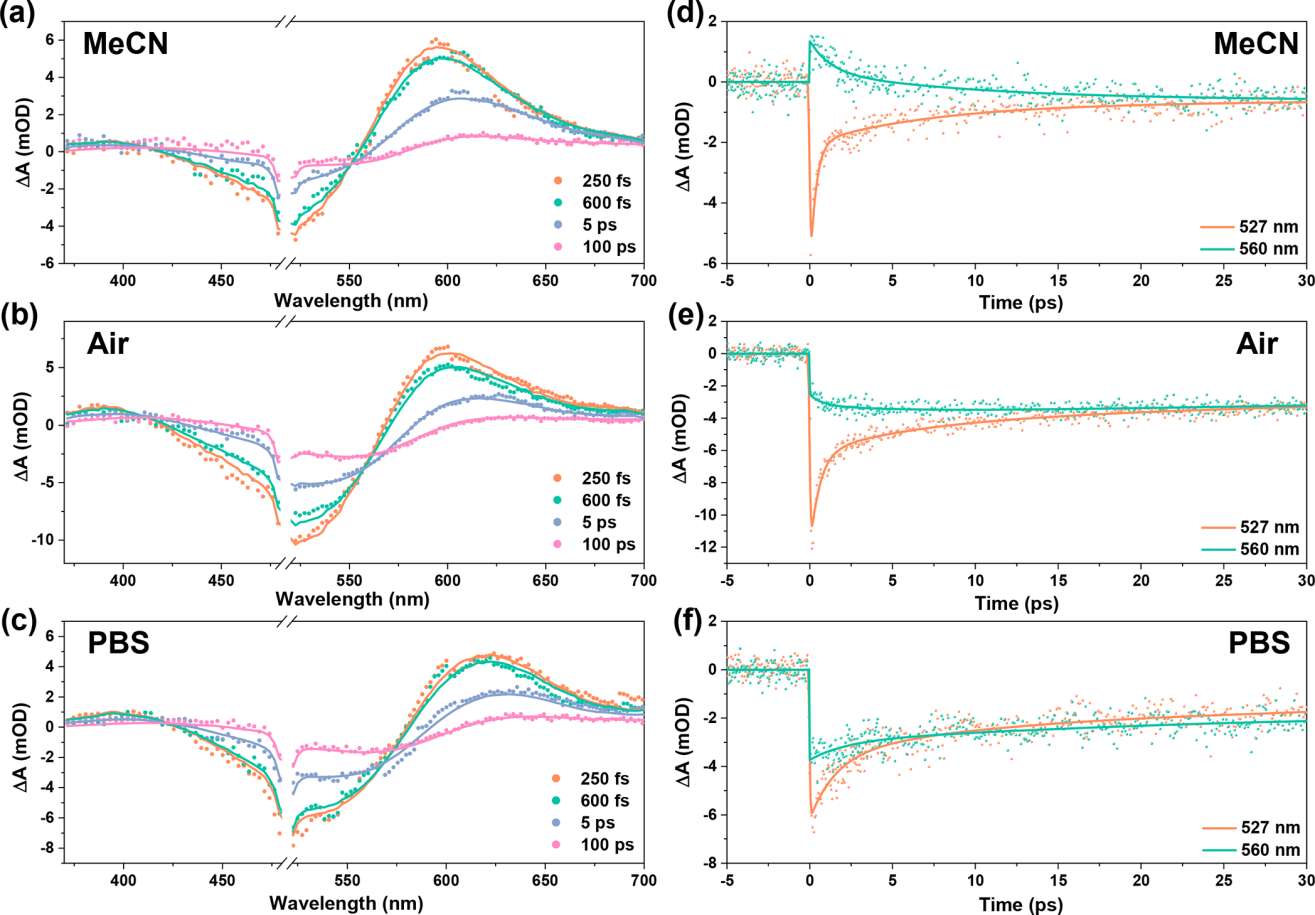
TA spectra after excitation at 500 nm (a–c) and kinetic traces (d–f) of NiO/P1 in dry MeCN (a,d), air with a relative humidity of 40% (b,e), and PBS (c,f). The lines indicate fits from target analysis.

To investigate the role of surface hydroxyl groups in the photodynamics in detail, femtosecond TA studies were performed on the same sample, in the order air–PBS–dry acetonitrile (MeCN) (Figure 3). Considering the difference in OH^–^ surface termination of NiO in aqueous and nonaqueous electrolytes^37^ and the equilibrium between surface-dissociated adsorbed and bulk water,^31,33^ it is reasonable to assume that the NiO surface in aqueous solution contains more hydroxyl groups than in air and especially in dry MeCN. The similar dynamics in PBS without and with prior N_2_ purging (Supporting Information Figure S4) exclude a role of dissolved CO_2_ or O_2_. The broad negative signal is due to the photoinduced ground-state bleach (GSB) of the P1 dye. The P1 excited state (P1*) is known to give a strong and broad positive absorbance above 550–560 nm and a weak absorbance around 410 nm.^44,49^ Due to hole injection from P1* into NiO, the intensity of P1* decreases, whereas the characteristic spectrum of P1^•–^ arises (positive bands around 420 and 610 nm).^44^ Photoinduced hole injection is known to cause a red shift in the TA spectrum due to overlapping signals of P1 GSB, P1*, P1^•–^, and Ni^3+/4+^^44,49−51^ (Supporting Information Figure S3). It is notable that the spectra for NiO/P1 in air and especially in PBS at early times (<1 ps) are red-shifted compared to those of NiO/P1 in MeCN, which can be explained by more photoinduced hole injection within the instrumental response time (IRT, 100–150 fs) in air/PBS. This can also explain the less intense negative signal for NiO/P1 in dry MeCN (Figure 3a), which is likely caused by a stronger P1* absorbance overlapping with the GSB.

Figure 3d–f compares the kinetic traces at 527 and 560 nm of NiO/P1 in dry MeCN, air, and PBS. The difference between the traces at 527 and 560 nm is significant and mainly caused by photoinduced hole injection. The 560 nm signal is due to both P1* absorption and GSB, while the 527 nm signal is mainly due to GSB.^44,49^ The decay at 560 nm is hence largely due to P1* decay via hole injection into the NiO, while the decay at 527 nm is mainly due to charge recombination following hole injection. It is important to distinguish between hole injection within the IRT and beyond, which is a well-known biphasic behavior.^44^ The difference between the traces at 527 and 560 nm until a few picoseconds is the largest in MeCN, intermediate in air, and the smallest in PBS, indicating more hole injection <IRT in air and in particular in PBS. A larger amount of surface hydroxyl groups hence implies extensive ultrafast photoinduced hole injection. The small difference between the 527 and 560 nm traces for NiO/P1 in PBS demonstrates only minor hole injection beyond the IRT, and the decay is hence mainly caused by charge recombination. In air and, in particular, in dry MeCN, hole injection also occurs beyond the IRT until a few picoseconds, while charge recombination occurs simultaneously and beyond.

**Figure 4:**
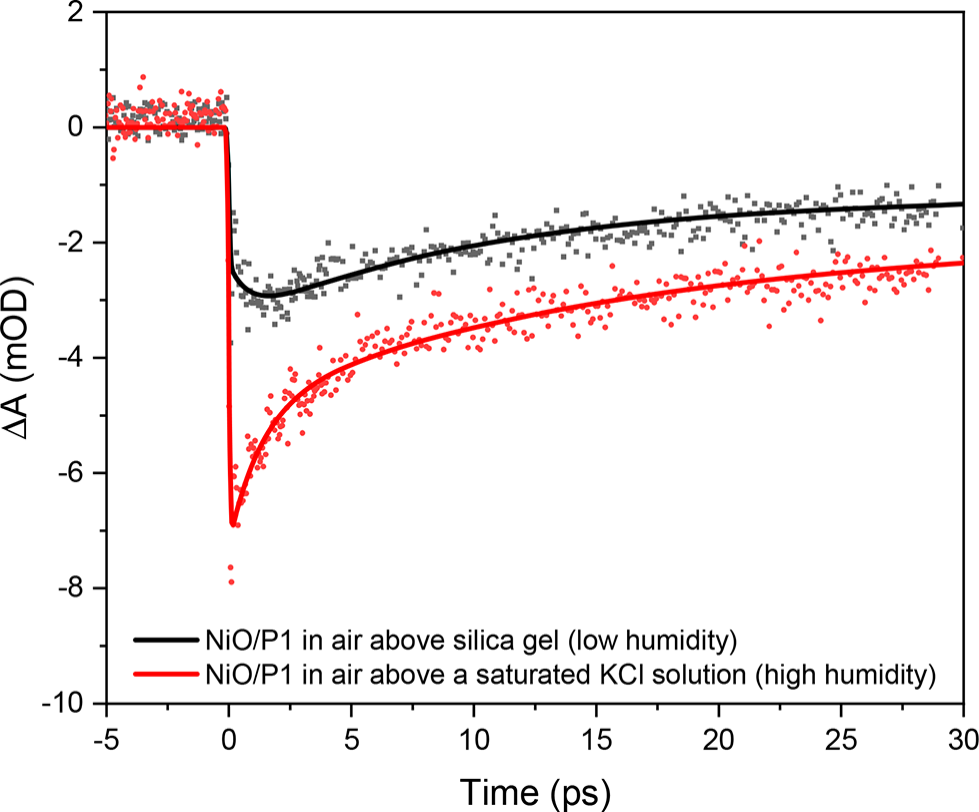
TA kinetic traces after excitation at 500 nm probed at 560 nm of NiO/P1 measured first in an air-filled sealed cuvette above silica gel and then above a saturated KCl solution, including fits from target analysis.

The difference between PBS and H_2_O environment (data in Supporting Information Figure S5) is minor, demonstrating that the effect of phosphate ions is small or even negligible and not responsible for the trends shown in Figure 3. To obtain further evidence that these differences are caused by H_2_O-induced surface hydroxyl groups, we have measured a new NiO/P1 layer in air, first with a low relative humidity and then with a high relative humidity, controlled by using silica gel or a KCl saturated solution in the air-filled sealed cuvette containing the NiO/P1 layer (see Supporting Information Figure S6–S7). The kinetic traces at 560 nm shown in Figure 4 clearly demonstrate different dynamics. In dry air, the initial <IRT rise in the negative signal is followed by a slower few picosecond rise, similar to that in Figure 3e. In contrast, in air with a high humidity, all transient signal at 560 nm develop within the IRT, similar to that in PBS or H_2_O (Figures 3f and S5). Figure 4 hence independently proves that a difference in quantity of surface hydroxyl groups is responsible for the trends shown in Figure 3.

### Surface Hydroxyl Groups Promote Charge Recombination

**Figure 5:**
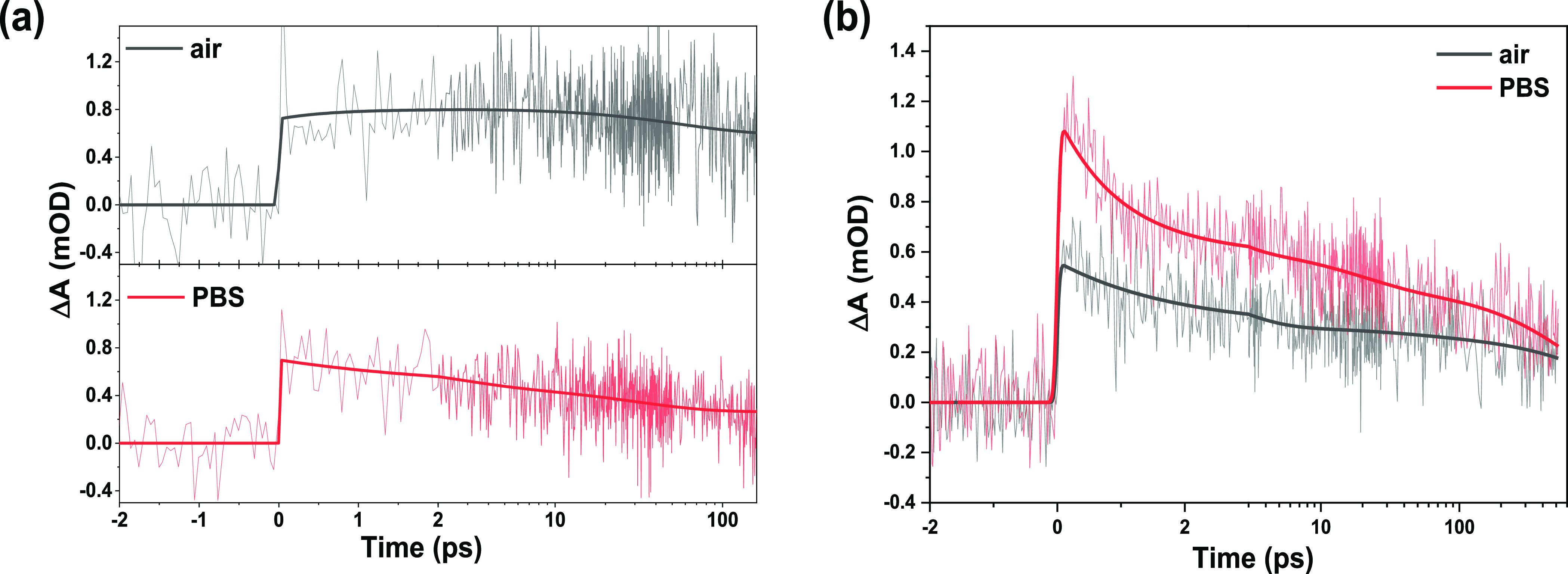
TA kinetic traces after excitation at 500 nm probed at 410 nm (a) and 813 nm (b) of NiO/P1 in air and PBS, including fits from target analysis.

**Figure 6:**
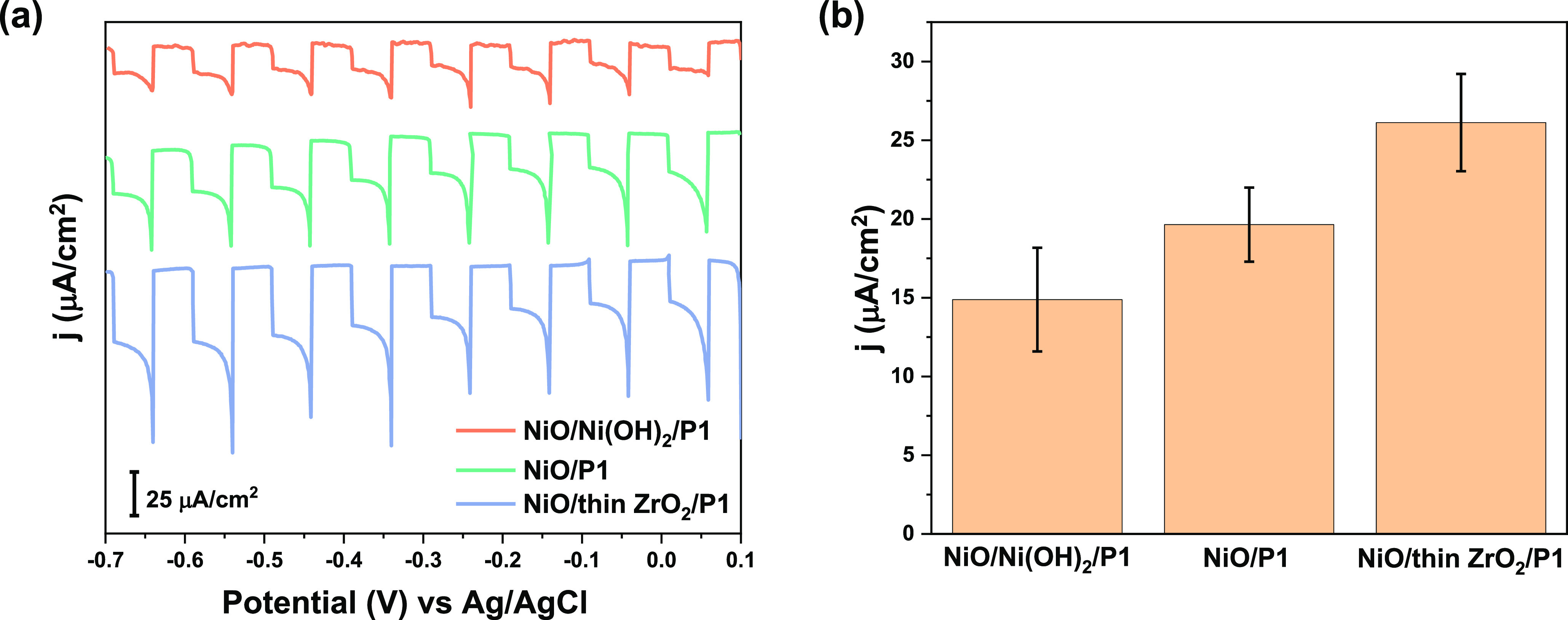
(a) PEC performance in 0.1 M PBS at pH = 7 using chopped illumination of 1 sun (AM 1.5G, >400 nm) of NiO/P1, NiO/Ni(OH)_2_/P1, and NiO/thin ZrO_2_/P1 photocathodes. (b) Average and errors of the photocurrent at −0.15 V vs Ag/AgCl of NiO/P1, NiO/Ni(OH)_2_/P1, and NiO/thin ZrO_2_/P1 photocathodes.

The decay of the TA band from 350–420 nm previously assigned to P1^•–^ is illustrative for charge carrier recombination.^44^Figure 5a shows the kinetic traces at 410 nm for P1/NiO in air and PBS, the transient signals in dry MeCN are very weak (Figure S9, likely due to the low amount of P1^•–^). In PBS, the signal seems to decay faster, suggesting faster charge recombination. P1^•–^ is known to also absorb around 610 nm.^44^ The trace at 610 nm clearly shows a slower decay in MeCN than in air and PBS (Figure S8, Supporting Information) demonstrating slower charge recombination in MeCN, but the difference between air and PBS is not obvious, possibly due to the overlap in signals. Traces in the blue and near-infrared regions are therefore compared instead (Figure 5b). Both the traces at 410 and 813 nm clearly show the fastest P1^•–^ decay in PBS, which could be due to 1) electron injection by P1^•–^ into the PBS and/or 2) fast charge recombination after hole injection promoted by the abundant surface hydroxyl groups. The lower photocurrent for NiO/Ni(OH)_2_/P1 relative to that of NiO/P1 (Figure 6, both measured in PBS) demonstrates that the second scenario is most likely. A possible reason for this fast charge recombination after hole injection is hole accumulation. Transition metal hydroxyl oxides are well-known supercapacitor materials, and they have the ability to store charges, either from the additional bias^51−53^ or from photogenerated charges.^47,54^ In addition, NiO is a p-type material but suffers from a poor hole mobility, which means that after ultrafast hole injection, most of the holes likely remain at, or close to the OH^–^-terminated surface. This is in agreement with literature studies reporting holes to be mainly pinned at the NiO surface,^55^ increasing the chance to recombine with P1^•–^.

To quantify effects of OH^–^ surface termination on hole injection and charge carrier recombination and account for the overlap in TA signals, target analysis has been performed based on the discussion above. The photophysical model used is shown in Figure S10 of the Supporting Information, and the results are provided in Table 1. Although this model is likely a simplification of the reality, it describes the TA data well. Hole injection (τ_1_) occurs the fastest in PBS (<IRT), followed by air (690 ± 12 fs) and dry MeCN (1270 ± 28 fs). Hole injection is typically biphasic, with the slower component occurring simultaneously to fast charge carrier recombination, which is combined in τ_2._ Finally, τ_3_ describes slower charge carrier recombination, which is clearly identified to be the slowest in MeCN and the fastest in PBS, while τ_4_ presents a minor nondecaying component.

**Table 1:** Lifetimes of NiO/P1 in Various Environments Obtained from Target Analysis, with the Species-Associated Spectra Provided in Figure S11 of the Supporting Information

environment	τ_1_ (fs)	τ_2_ (ps)	τ_3_ (ps)	τ_4_ (ps)
MeCN	1270 ± 28	11 ± 0.2	370.8 ± 11.4	
Air	690 ± 12	5.7 ± 0.1	56.1 ± 1.4	∞
PBS	within IRT	2.2 ± 0.1	30.6 ± 0.4	∞

Now that we have unraveled the dual role of surface hydroxyl groups in the photodynamics, we will discuss the effect of the surface composition on the PEC performance. In Figure 6, we compare the photocurrent traces of (i) NiO/P1, (ii) NiO/P1 with an OH^–^-rich surface by introducing a thin layer of Ni(OH)_2_, and (iii) NiO/P1 with an OH^–^-poor surface by adding a thin ZrO_2_ layer (Figure 6) in between the dye and the NiO surface. The surface OH^–^ versus O^2–^ ratio for these samples has been estimated using XPS and is further detailed in Figure S12 of the Supporting Information. The UV–vis absorbance spectra (Figure S13 Supporting Information) show no significant differences in light adsorption by P1. In addition, the intermediate PL quenching of NiO/thin ZrO_2_/P1 relative to that of NiO/P1 and P1/quartz confirms the existence of some ZrO_2_ at the interface (Figure S14 Supporting Information). NiO/thin ZrO_2_/P1 shows the highest photocurrent, NiO/P1 shows an intermediate behavior, and NiO/Ni(OH)_2_/P1 exhibits the lowest photocurrent. Another noteworthy photocurrent feature of NiO/Ni(OH)_2_/P1 is the significantly lower transient photocurrent after initiating illumination. This transient photocurrent usually indicates (capacitive) charge storage.^47,56^ The decrease in intensity of the capacitive current, without an increase in steady-state photocurrent, indicates that these accumulated charges likely recombine rather than being harvested, which is another indication that OH^–^ groups at the NiO surface promote charge recombination.

**Figure 7:**
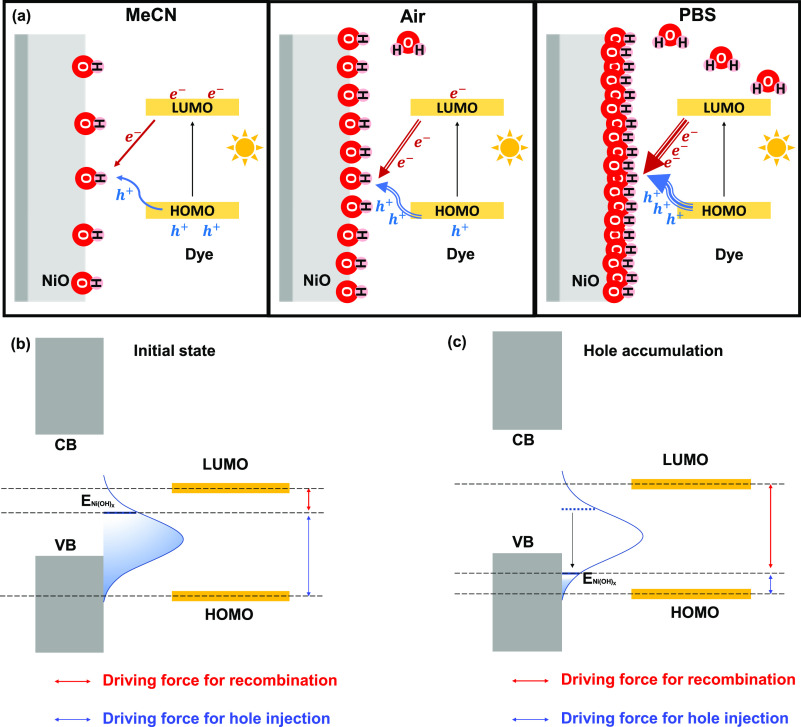
(a) Simplified schematic diagram of the dual role of surface hydroxyl groups, promoting both photoinduced hole injection and charge recombination. (b) Energy diagram before hole injection and (c) after surface hole accumulation.

Figure 7 illustrates the dual role of surface OH^–^ groups unraveled in the present work, of which the amount has been tuned from low to high by changing the environment of the photoelectrode from dry MeCN to air and PBS or through the introduction of compositional changes. A low quantity of OH^–^ groups not only leads to a relatively low rate of photoinduced hole transfer (the blue arrow) but also prevents significant transfer of electrons from photoactivated dye molecules, lowering the rate of charge recombination. Passivation of the NiO surface by, for example, Al, Al_2_O_3_, or ZrO_2_ will reduce the amount of OH^–^, which is beneficial for the photocurrent in aqueous conditions (Figure 6 for ZrO_2_)^26^ but was previously observed to be disadvantageous for the performance of solar cells (i.e., MeCN-based electrolyte),^20,21^ suggesting that the amount of surface OH^–^ should not be too low. An aqueous electrolyte (PBS) introduces a significant quantity of surface OH^–^ groups, which promote both hole transfer (indicated by the increased thickness of the blue arrow) and charge recombination (indicated by the thickness of the red arrow).

NiO is known to have a poor hole mobility, while Ni(OH)_2_ is a good hole storage material.^47,52−54^ In PBS, injected holes are likely pinned at the NiO surface due to the sluggish hole mobility,^55^ promoting charge recombination with the electrons of the reduced dye molecules.

This dual role of surface hydroxyl groups can be understood by the adaptive junction theory that Boettcher proposed for semiconductor–electrocatalyst interfaces.^57,58^ In this theory, the transition metal hydroxide or oxyhydroxide is a redox-active ion-permeable electrocatalyst, which can form an adaptive semiconductor/electrocatalyst junction. When holes are injected into this transition metal hydroxide thin layer, the Fermi level shifts down to the valence band of the semiconductor due to surface hole accumulation (Figure 7), reducing the driving force for hole injection from the P1 HOMO. A low amount of surface hydroxyl groups causes less fast hole injection and may explain the biphasic photoinduced hole injection typically observed in air^44^ and also in the present work (<IRT and 690 ± 12 fs). More surface hydroxyl groups imply more *E*_Ni(OH)_*x*__ at the initial state, which can accept the holes from the dye much faster, explaining the <IRT hole injection in PBS. Considering the low hole mobility of NiO,^55^ this ultrafast hole injection will likely promote hole accumulation and shift *E*_Ni(OH)_*x*__ down, resulting in a larger driving force for charge recombination in PBS. Considering the similar character of transition metal oxides and hydroxides, the dual function of surface OH^–^ groups might also explain the contradictory results for TiO_2_, Fe_2_O_3_, and other metal oxide photoelectrodes.^19−23,26,27^ Our work illustrates the importance of balancing the number of surface hydroxyl groups on an oxide semiconductor surface to control photoinduced charge separation and recombination and to realize efficient solar fuel devices.

## Conclusions

In summary, we provide direct evidence of the dual function of an OH^–^-terminated NiO surface. Photoinduced hole injection is accelerated by an increased quantity of Ni–OH; however, the injected holes are likely pinned at the surface due to the hole storage ability of Ni(OH)_2_ and the low hole mobility of bulk NiO, promoting charge recombination with electrons from the reduced dye molecules. The dependency of the hole injection and recombination rate on the working environment can explain why photoelectrodes in an aqueous electrolyte show inferior performance relative to similar photoelectrodes in an acetonitrile-based electrolyte. The dual function of surface OH^–^ groups could also explain the conflicting results in the literature, not only for NiO but also for other metal oxide-based photoelectrodes. We believe that our results illustrate the importance of balancing the number of surface hydroxyl groups on an oxide semiconductor to optimize photoinduced charge separation and recombination and guide the design of efficient solar devices.

## Supporting Information Available

Experimental section, SEM images, X-ray diffraction patterns, UV–vis spectra, TA data and photophysical model used for target analysis, species-associated spectra, XPS spectra, and PL decay data (PDF)
