# Supplementary material for: Dual Role of Surface Hydroxyl Groups in the Photodynamics and Performance of NiO-Based Photocathodes
Source: J Am Chem Soc. 2022 Jun 8;144(24):11010–8. doi: 10.1021/jacs.2c04301 (PMC9228059; doi:10.1021/jacs.2c04301)
Supplement: Supplementary file 1 — ja2c04301_si_001.pdf [file ja2c04301_si_001.pdf]

Dual Role of Surface Hydroxyl Groups in the Photodynamics and Performance of NiO-Based Photocathodes

Supporting information

*Kaijian Zhu,[†] Sean Kotaro Frehan,[†] Guido Mul,[†] Annemarie Huijser *[†]*

[†]PhotoCatalytic Synthesis Group, MESA+ Institute for Nanotechnology, University of Twente, P.O. Box 217, 7500 AE Enschede, the Netherlands

*Corresponding Author

Annemarie Huijser
E-mail: j.m.huijser@utwente.nl

Experimental Section

Sample Preparation

The nanoporous NiO films were prepared following a procedure reported in the literature, in air with a relative humidity of 40% to 50%.[1] The as-prepared NiO films were dipped into P1 dye (4-(bis-4-(5-(2,2-dicyano-vinyl)-thiophene-2-yl)-phenyl-amino)-benzoic acid, purchased from Dyenamo, Sweden) dissolved in ethanol (Supelco, >99.9 %) for around 16 h. ZrO_2 films were also prepared according to an earlier reported procedure.[2] The zirconium precursor solution was prepared by adding 40 mM zirconium butoxide (Sigma-Aldrich, 80 wt. % in 1-butanol) into 1-butanol solution (Sigma-Aldrich, 99.9%). The as-prepared photoelectrodes were dipped into the precursor solution and kept at 70 °C for different times (15 min. for thin ZrO_2 and 1 hour for thick ZrO_2). Before P1 dye deposition, the layers were annealed at 475 °C for 30 min in air.

The $Ni(OH)_2$ layers were prepared by a chemical bath deposition method. The precursor solution was prepared by dissolving 0.03 M $Ni(NO_3)_2 \bullet 6H_2O$ (Sigma-Aldrich, 99.999%), 0.08 M urea (Sigma-Aldrich, >99%) and 0.3 M ethanolamine (Sigma-Aldrich, >99%) into Milli-Q water. The as-prepared film electrode was put into the solution and kept there at 90 °C for 10 min. Finally, the film was taken out and rinsed by Milli-Q water.

Characterization

The crystal structures were characterized by X-ray diffraction (Bruker D2, Cu Kα source). The UV-vis spectra were measured in transmission mode using a ThermoSci EVO600 spectrometer. The valence states of the surface were characterized by X-ray Photoelectron Spectroscopy (XPS, PHI Quantera SXM).

Time resolved photoluminescence spectroscopy (TRPL)

The time-resolved photoluminescence was measured by a streak camera setup (Hamamatsu, C10910), using the attenuated output of a Fianium laser (FP-532-1-s, center wavelength 532 nm, pulse duration of 300 fs, 80.37 MHz repetition rate), which was focused using a quartz lens with 50 mm focal length onto the samples in a quartz cuvette (Hellma, 10 mm optical path length) filled with air, 0.1 M phosphate buffer solution (PBS, pH=7) or anhydrous acetonitrile (Sigma-Aldrich, 99.8 %). The photoluminescence was collected using two 2-inch diameter 50 mm focal length glass lenses and focused on the input of a spectrograph (Acton SP2300, Princeton Instruments, slit width set at 100 mm) using a grating with 50 lines/mm blazed at 600 nm. The output of the spectrograph was sent to the photocathode of the streak camera. Quartz (Uqg optics) was used as the substrate to avoid background photoluminescence signal. Before each series of experiments, the spectral calibration was checked using a Hg/Ar calibration lamp (Oriel, LSP035) and adapted if necessary.

Femtosecond transient absorption spectroscopy (fs TA)

The fs TA setup was described in detail in our previous paper.[1] The 800 nm pulses at 5 kHz repetition rate with a pulse duration of 35 ± 1 fs FWHM were generated by a Ti:Sapphire amplifier (Coherent, Legend Elite). A 90:10 beam splitter was used to split the 800 nm output into two beams. The 500 nm pump beam was generated by sending the major part of the 800 nm beam into an optical parametric amplifier (Coherent, Opera). The remaining part of the 800 nm beam was sent through a mechanical delay stage and after further attenuation focused into a CaF_2 crystal (Newlight Photonics, 3 mm thickness) mounted on a continuously moving stage. An OD 3 near-IR filter (NENIR30B) and OD 1 near-IR filter (NENIR10B) from Thorlabs instead of the 700 nm short-pass filters [(1)] were used to extend the probe window from 700 nm to 850 nm. The polarizations of the pump and probe beams were set at 54.7° magic angle. The differential absorbance between pump on and off was obtained by chopping the pump beam at 2.5 kHz. The time resolution is ca. 100−150 fs. The samples were mounted on a continuously moving stage with a velocity of ca. 1 mm/s to refresh the measurement area regularly and avoid potential charge accumulation and photodegradation. Samples were checked for photodegradation by comparing UV–vis absorbance spectra before and after the TA measurements

and no changes were observed. The pump power was kept relatively low (<5 ×10^{14} photons/(cm^2 pulse)) and verified to be in the linear regime. The TA experiments were carried out on the dry film in a quartz cuvette (Hellma, 5 mm optical path length) in air, PBS, and dry acetonitrile. Before the measurements, the film and cuvette were blown by N_2. To avoid sample variation, all comparative experiments were performed on the same NiO photocathode. The data were analysed using the open-source program Glotaran.[3]

Photoelectrochemical measurements

Photoelectrochemical (PEC) properties were measured in a three-electrode cell using an VersaSTAT 3 Potentiostat under illumination by a solar simulator with AM 1.5G filter (Newport, 1 sun intensity) and a filter cutting off UV below 400 nm. An Ag/AgCl electrode and gold wire were used as the reference electrode and counter electrode. The PBS electrolyte was degassed by N_2 for more than 20 min. prior to the measurements. Scans were performed from high to low potential, with a scan rate of 5 mV/s.

Supplementary results

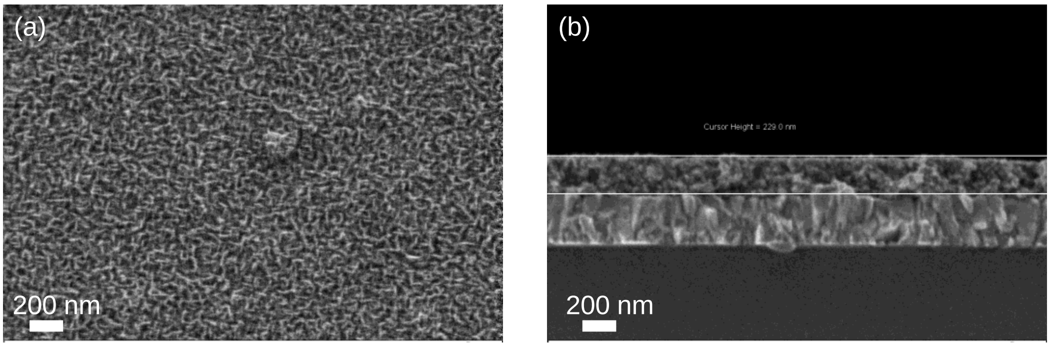

Figure S1. (a) Surface and (b) cross-section scanning electron micrographs of NiO on fluorine-doped tin oxide (FTO)/glass substrates.

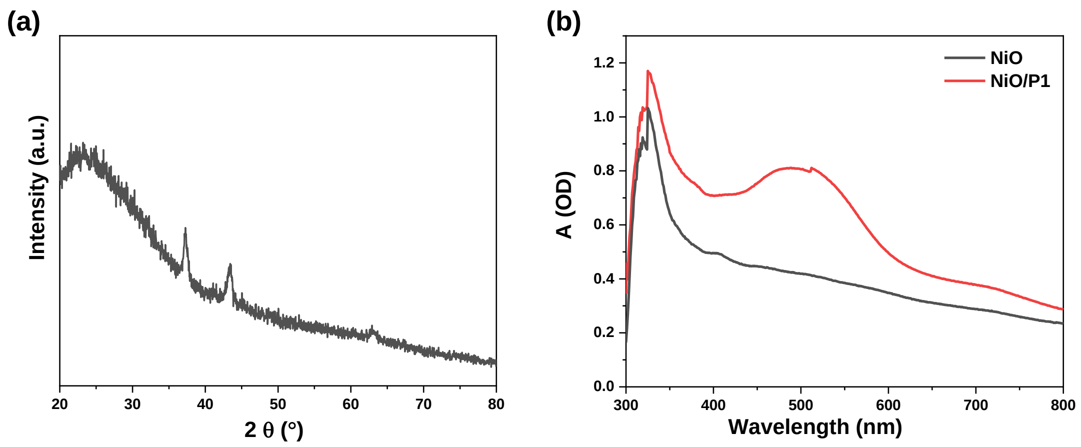

Figure S2. (a) XRD patterns of NiO on amorphous glass; (b) UV-vis absorbance spectra of NiO with and without P1 dye.

Fig. S1 presents the surface and cross-section electron micrographs of NiO on FTO/glass, showing a homogeneous nanoporous layer with a thickness of ca. 230 nm. Fig. S2a shows the XRD peaks of NiO around 37° and 43°, which can be assigned to (111) and (200) facets of NiO.[1] The featureless band around 22° is due to the amorphous glass substrate. Fig. S2b shows the UV-vis absorbance spectra of NiO with and without P1 dye, with NiO/P1 showing maximum absorbance around 500 nm.

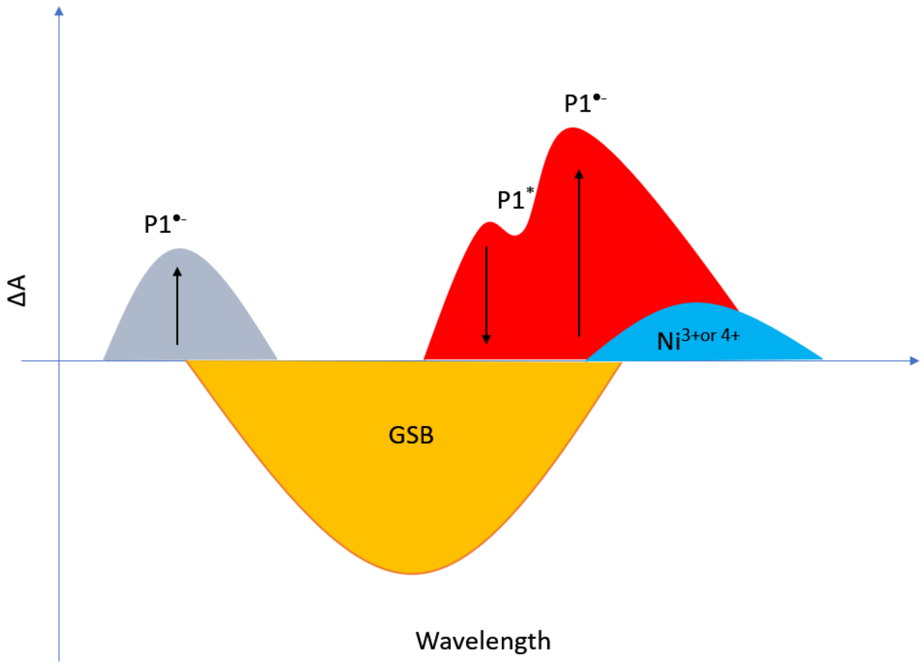

Figure S3. TA signals of NiO/P1 photoelectrodes, the arrows indicate the spectral evolution due to hole injection by P1* into the NiO.

Fig. S3 illustrates the overlap in TA signals of NiO/P1 photoelectrodes, with a broad ground state bleach (GSB) due to absorption by the P1 dye. Photoexcitation gives the excited state P1*, with a strong absorption around 560 nm and a weak absorption around 410 nm (in MeCN).[4] The signal of P1* of NiO/P1 might be red-shifted due to electronic coupling.[5] After hole injection from P1* into NiO, the formed $P1^{\bullet-}$ shows absorption bands around 610 nm and 420 nm. Hammarström et. al. observed that Ni^{3+} or Ni^{4+} sites give rise to absorption >600 nm.[6]

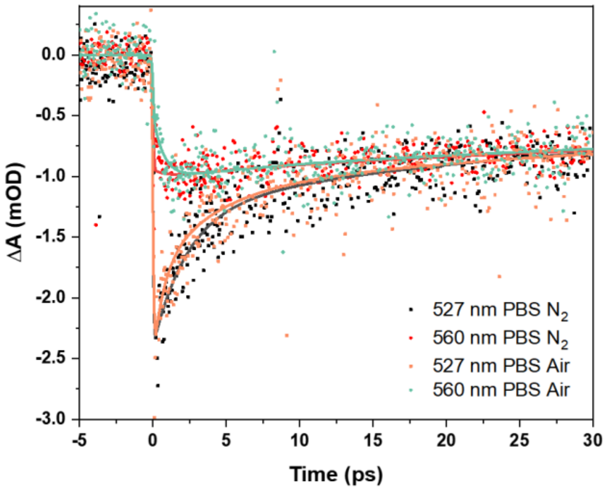

Figure S4. Kinetic traces of the same NiO/P1 layer in PBS without and with prior N_2 bubbling for 1 h, including fits from target analysis as solid lines.

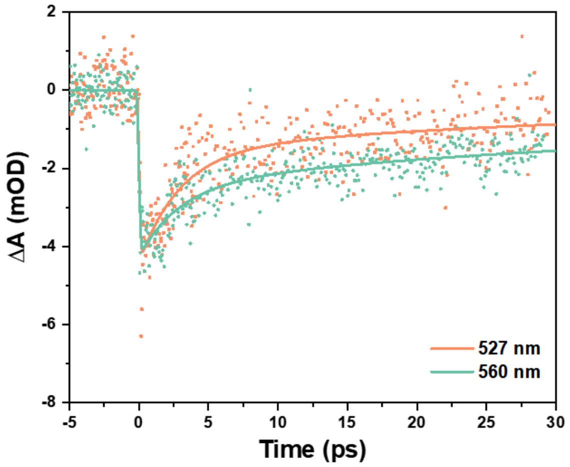

Figure S5. Kinetic traces of the NiO/P1 layer in milli-Q H_2O, including fits from target analysis as solid lines.

Fig. S4 shows selected kinetic traces for the same Ni/P1 sample in PBS without and with prior N_2 bubbling for 1 h to remove dissolved O_2 and CO_2. The minor difference between the fits at early times is likely due to a slightly shifted P1* spectrum resulting from fitting in Glotaran for both cases. The similarity in data shows that bubbling with N_2 for 1 h does not alter the photodynamics, excluding a significant effect of dissolved O_2 or CO_2. The little stronger GSB relative to the P1* signal (560 nm) compared to Fig. 3f in the main text is likely due to small variations in absorbance of different samples. Fig. S5 shows selected kinetic traces for Ni/P1 in milli-Q H_2O, with the only minor difference with PBS (Fig. 3 main text) excluding a significant effect of phosphate ions on the photodynamics.

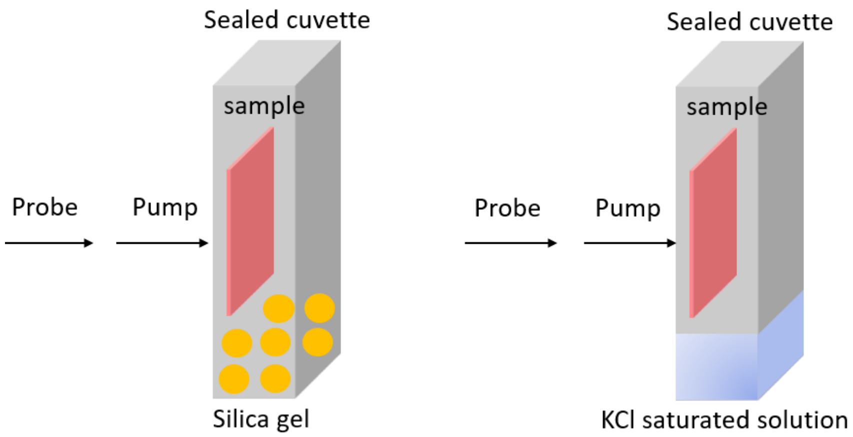

Figure S6. Illustration of the transient absorption measurements in different environments.

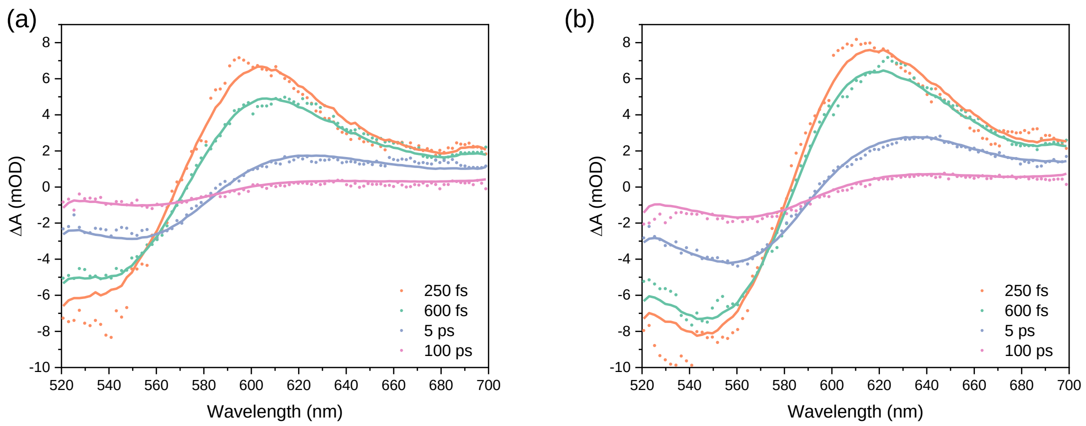

Figure S7. Transient absorption spectra after excitation at 500 nm of NiO/P1 in (a) air dried with silica gel and (b) air in equilibrium with a KCl saturated solution. The solid lines present fits from target analysis. The deviation between fit and the data <540 nm at 250 fs is due to the chirp correction.

Fig. S6 illustrates the experimental setup used for investigating the role of air with different relative humidity on the photodynamics. Silica gel is usually used in a desiccator to keep the environment dry. KCl saturated solution can be used to control the air relative humidity to more than 80%.[7] Fig. S7 presents the transient absorption spectra for these two cases including fits from target analysis (Fig. S10) as solid lines.

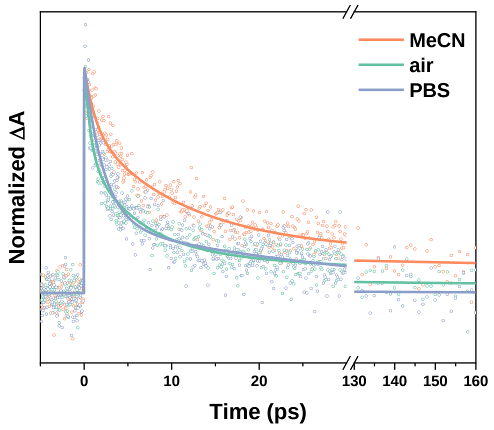

Figure S8. Kinetic traces at 610 nm of NiO/P1 in dry MeCN, air and PBS, including fits from target analysis as solid lines.

Fig. S8 shows the kinetic traces at 610 nm. It is obvious that NiO/P1 in dry MeCN shows a much slower decay compared with in air and PBS. The difference between air and PBS is less obvious, but clearly visible around 813 nm (Fig. 5b main text).

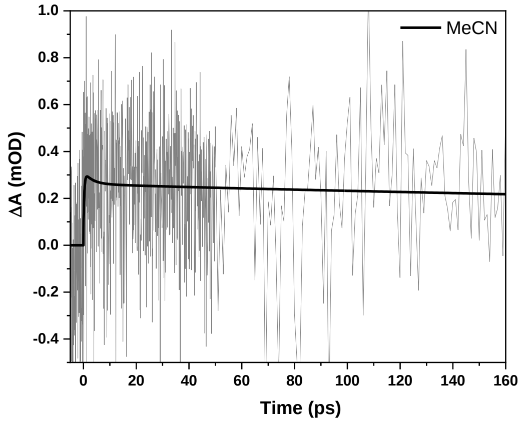

Figure S9. Kinetic traces at 410 nm of NiO/P1 in MeCN, including fits from target analysis as solid lines.

Fig. S9 shows the kinetic trace at 410 nm of NiO/P1 in MeCN, previously assigned to $P1^{\bullet -}$ and illustrative for charge carrier recombination.[4] The weaker signal relative to NiO/P1 in air or PBS (main text Fig. 5a) is likely caused by slower hole injection assigned to the low abundance of surface OH^- groups. We therefore neglect $P1^{\bullet -}$ formed during the IRT in MeCN in the model used for target analysis (Fig. S10, please see our recent publication[1] for further motivation of this photophysical model). In contrast, most of hole injection takes place within the IRT for NiO/P1 in PBS. A relatively low concentration of (surface) Ni^{3+} and Ni^{4+} giving rise to signal >600 nm is observed in MeCN. The obtained species associated spectra are shown in Fig. S11 and the obtained lifetimes are presented in Table 1 of the main text.

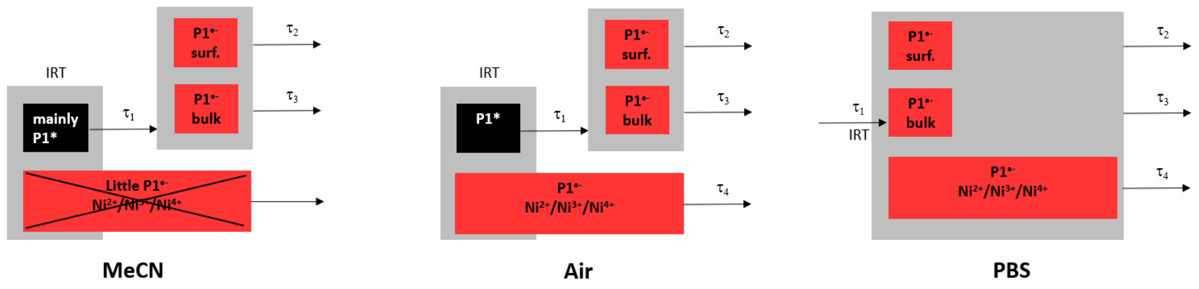

Figure S10. Photophysical model used for target analysis of NiO/P1 in dry MeCN (left), air (middle) and PBS (right). IRT = instrumental response time (100-150 fs).

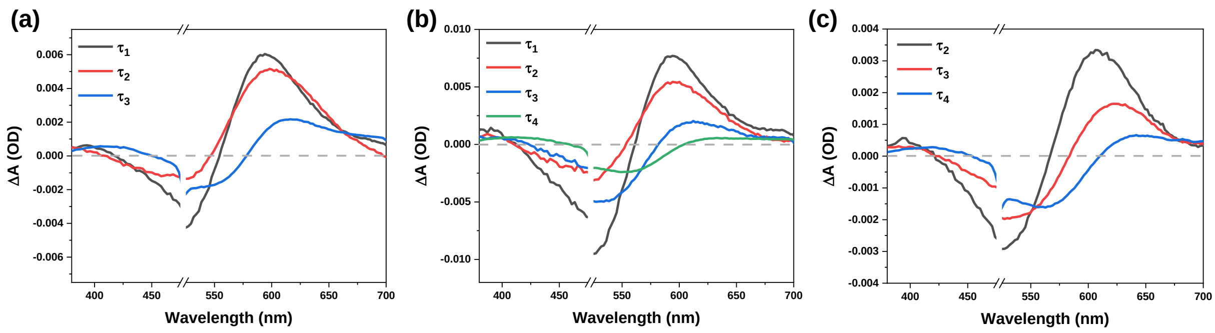

Figure S11. Species associated spectra (SAS) obtained from target analysis for NiO/P1 in (a) MeCN, (b) air and (c) PBS.

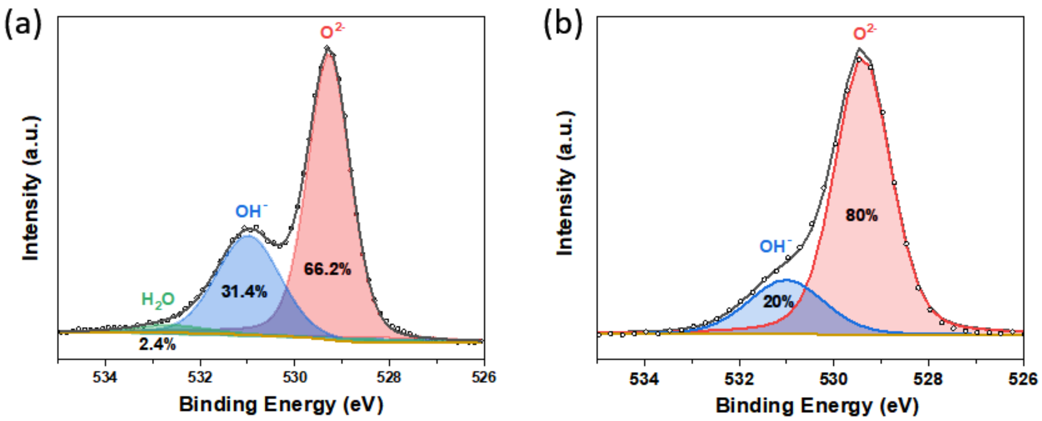

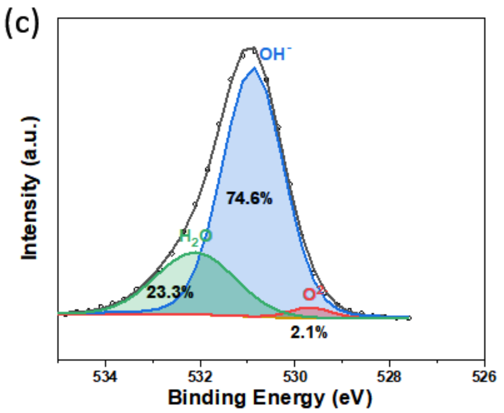

(d)

sample	OH^- : O^{2-}
NiO	2
NiO/ZrO_2	0.25
$NiO/Ni(OH)_2$	36

Figure S12. O 1s XPS spectra of NiO (a), NiO/ZrO_2 (b) and $NiO/Ni(OH)_2$ (c) and ratio of OH^- vs. O^{2-} on the surface (d).

Fig. S12 shows the O 1s XPS spectra of NiO (a), NiO/ZrO_2 (b) and $NiO/Ni(OH)_2$ (c) and the ratio of OH^- vs. O^{2-} on the surface (d), indicating that the quantity of surface hydroxyl group is the highest for $NiO/Ni(OH)_2$ and lowest for NiO/ZrO_2.

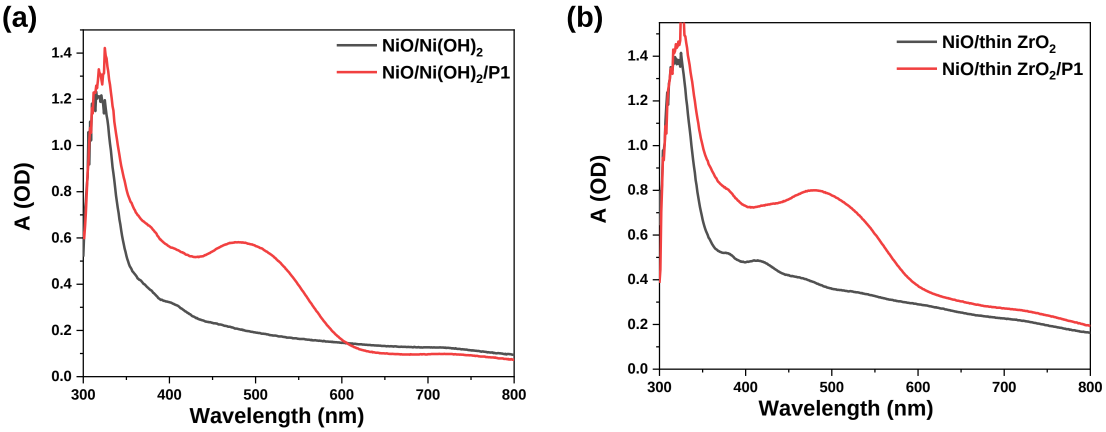

Figure S13. UV-vis absorbance spectra of NiO/$Ni(OH)_2$/P1 and NiO/ZrO_2/P1.

Fig. S13 shows the UV-vis absorbance spectra of NiO/$Ni(OH)_2$ (a) and NiO/thin ZrO_2 (b) with and without P1 dye. Alike for the NiO-based electrode (Fig. S1b), also here the typical P1 absorption centered around 500 nm is observed.

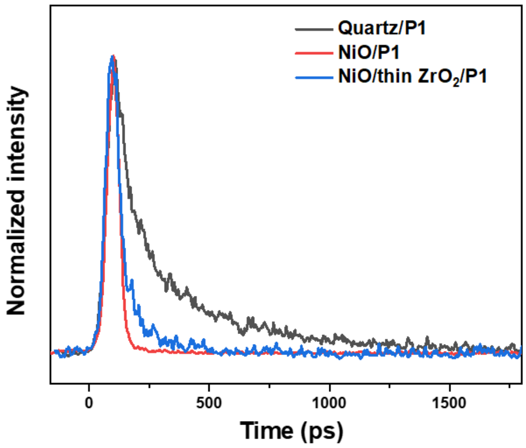

Figure S14. Normalized photoluminescence decay at 670 nm of different samples in air.

Fig. S14 shows the PL decay in air, for quartz/P1, NiO/P1 and NiO/thin ZrO_2/P1. The PL signal of NiO/P1 decays very fast compared with quartz/P1 due to ultrafast hole injection (<10 ps) from P1* into the NiO. Intermediate behavior is observed for NiO/thin ZrO_2/P1, indicative for some hole injection.

References

(1) Zhu K.; Frehan S.K.; Jaros A.M.; O’Neill D.B.; Korterik J.P.; Wenderich K.; Mul G.; Huijser A. Unraveling the Mechanisms of Beneficial Cu-Doping of NiO-Based Photocathodes. *J. Phys. Chem. C*, **2021**, *125*, 16049-16058.

(2) Kim B.-S.; Park J.-Y.; Kim C.-S.; Kim S.-B.; Song D.-K.; Jang H.-D.; Lee S.-E.; Kim T.-O. Zirconium oxide post-treatment for TiO_2 photoelectrodes in dye-sensitized solar cells. *Electrochim. Acta* **2015**, *174*, 502-507.

(3) Snellenburg, J., Laptenok, S., Seger, R., Mullen, K.M., Van Stokkum, I. Glotaran: A Java-Based Graphical User Interface for the R Package TIMP. *J. Stat. Soft*. **2012**, *49*, 1-22.

(4) Zhang L.; Boschloo G.; Hammarström L.; Tian H. Solid State P-Type Dye-Sensitized Solar Cells: Concept, Experiment and Mechanism. *Phys. Chem. Chem. Phys.* **2016**, *18*, 5080-5085.

(5) Qin P.; Zhu H.; Edvinsson T.; Boschloo G.; Hagfeldt A.; Sun L. Design of an Organic Chromophore for P-Type Dye-Sensitized Solar Cells. *J. Am. Chem. Soc.* **2008**, *130*, 8570-8571.

(6) D'Amario L.; Föhlinger J.; Boschloo G.; Hammarström L. Unveiling Hole Trapping and Surface Dynamics of NiO Nanoparticles. *Chem. Sci.* **2018**, *9*, 223-230.

(7) O'Brien F.E.M. The Control of Humidity by Saturated Salt Solutions. *J. Sci. Instrum.* **1948**, *25*, 73.
